# Human pluripotent stem cells for the modelling of retinal pigment epithelium homeostasis and disease: A review

**DOI:** 10.1111/ceo.14128

**Published:** 2022-07-11

**Authors:** Jenna C Hall, Daniel Paull, Alice Pébay, Grace E. Lidgerwood

**Affiliations:** ^1^ Department of Anatomy and Physiology The University of Melbourne Parkville Victoria Australia; ^2^ The New York Stem Cell Foundation Research Institute New York New York USA; ^3^ Department of Surgery, Royal Melbourne Hospital The University of Melbourne Parkville Victoria Australia

**Keywords:** human pluripotent stem cells, retinal disease, disease modelling, retinal pigment epithelium

## Abstract

Human pluripotent stem cells (hPSCs), which include induced pluripotent stem cells and embryonic stem cells, are powerful tools for studying human development, physiology and disease, including those affecting the retina. Cells from selected individuals, or specific genetic backgrounds, can be differentiated into distinct cell types allowing the modelling of diseases in a dish for therapeutic development. hPSC‐derived retinal cultures have already been used to successfully model retinal pigment epithelium (RPE) degeneration for various retinal diseases including monogenic conditions and complex disease such as age‐related macular degeneration. Here, we will review the current knowledge gained in understanding the molecular events involved in retinal disease using hPSC‐derived retinal models, in particular RPE models. We will provide examples of various conditions to illustrate the scope of applications associated with the use of hPSC‐derived RPE models.

## 1 INTRODUCTION

In 2007, it was demonstrated that human adult somatic fibroblasts can be reprogrammed to a stem cell state through the overexpression of four pluripotent transcription factors, octamer‐binding transcription factor (OCT3/4), sex‐determining region Y‐box 2 (SOX2), myc‐proto‐oncogene (C‐MYC) and Kruppel‐like factor 4 (KLF4).^1^ The resulting induced pluripotent stem cells (iPSCs) exhibit the morphology and cell marker genes of human embryonic stem cells (hESCs).^1^ The advent of human pluripotent stem cells (hPSCs) has led to the development of a ‘disease‐in‐a‐dish’ concept, allowing the study of molecular and cellular mechanisms using relevant human cells of interest in vitro. Combining hPSC models with genome editing technology, gene regulation analysis and phenotypic analysis now provides powerful tools to interrogate events of human development, physiology and disease. The eye and the retina are no exception to this expanding field of research, with hPSCs having already been used to recapitulate events of development, normal physiology and pathophysiology including of the retinal pigment epithelium (RPE). The role of the RPE is central to many retinal diseases, from inherited retinal diseases (IRD) to age‐related macular degeneration (AMD). The use of hPSC‐derived retinal pigment epithelium (RPE) models do not yet recapitulate the complex neuroanatomy of the retina, but the culture of this specific cell type allows the precise study of a single layer of the retina that is affected in various retinal conditions. This subsequently enables the development of novel methodologies for testing therapeutic efficacy of compounds in retinal diseases, an important consideration for drug trials, as preliminary screens of patient personalised RPE could provide greater insight into candidates for clinical trials.

## 2 THE RETINAL PIGMENT EPITHELIUM

The RPE lines the back of the retina as a layer of pigmented, post‐mitotic and polygonal cells.^2^ The RPE has many fundamental functions that ensure the homeostasis and functioning of the retina, including: phagocytosis of shed photoreceptor outer segments, secretion of proteins for paracrine cellular communication, active transport of biomolecules, ion homeostasis, isomerization of all‐trans‐retinol to 11‐cis retinal, and light absorption in melanosomes. Although not sensory cells themselves, the RPE cells are involved in intimate bioprocessing with photoreceptors, providing a critical role in their structural and functional viability.^3^ The RPE is the most efficient phagocyte in the body, removing 10% of the length of the photoreceptor in outer segments daily.^4^ These photoreceptor outer segments are phagocytosed, metabolically processed, and secreted through the Bruch's membrane into the choroidal circulation.^5^ The morphologically polygonal RPE cells form tight junctions, creating a diffusion‐impermeable barrier. Thus, the RPE must actively transport glucose and other nutrients between the basolateral choroid and the apically embedded photoreceptors, allowing visual processing to occur.^6^ It is unsurprising that RPE dysfunction is connected to severe vision loss as its health and function is essential to photoreceptor survival and maintaining the balance of ions, water and pH within the subretinal space.

## 3 CURRENT MODELS OF RPE, ADVANTAGE OF HPSC DERIVED MODELS

To adequately capture the underlying molecular features of retinal diseases and subsequently elucidate potential therapies, it is essential to first establish appropriate models using human cells that recapitulate the disease phenotype. However, retinal diseases are challenging to model in vitro due to the paucity of access to human retinal tissue. As an alternative to a diseased human eye, given their genetic similarity to humans and parallel anatomical features, primate models have been developed, though are expensive to acquire and maintain. Comprehensive housing requirements and the need for highly experienced staff make them beyond reach of many research laboratories.^7^ Rodents are a cost effective alternative, and commonly used to investigate diseases of the optic nerve and the retina.^8^ Due to numerous orthogonal genes between rodent and human genomes, genetic studies in rodents are proving to be particularly enlightening with respect to disease‐causing variants and pathological mechanisms. Furthermore, laboratory mice live for only a handful of years and often develop diseases that can take years to advance in humans.^7^ Anatomically, rodent retinas differ from humans', for example, rodent optic nerves are superior colliculus making their eyes easily surgically accessible.^7^ Rodents do not have a macula or a fovea, the site of trauma in many human macular diseases in humans, and up to 90% of rodent optic nerve axons decussate to the opposite side of the brain as compared to humans.^9^ Approximately, 1% of human genes have no identifiable mouse homologues,^10^ thus primary cultures from human cadavers are a popular method for studying ocular disease in vitro. However, primary tissues begin to degrade following death of the donor.^11^ Therefore, swiftly obtaining and culturing primary tissue is imperative. Since it is logistically difficult to predict death and process a sample before degeneration, this medium of study is also limiting. While immortalised cell lines overcome this issue, a number of established lines fail to exhibit the morphological and functional characteristics of the native tissue or have an abnormal karyotype.^12^ The culture of hPSC‐derived RPE cells enables unrestricted access to the cells that comprise the RPE layer as it exists in vivo. Furthermore, genome editing methods such as CRISPR/Cas systems, can be used to generate isogenic hPSC lines that differ only at specific loci, thus the effects of chosen genetic variants can be precisely interrogated,^13^ for instance to uncover downstream changes in gene expression caused by a disease‐associated mutation,^14^, ^15^ or reveal the influence of a specific variant on a disease phenotype.^16^

## 4 DIFFERENTIATING HIPSCS TO RPE

The simplest way of producing a somatic cell from a pluripotent stem cell is routinely referred to as spontaneous differentiation. Pluripotent stem cells move out of pluripotency and down the developmental path of one of the three germ layers: ectoderm, endoderm or mesoderm.^17^ Spontaneous differentiation uses adherent or suspension culturing methods and relies on the inherent cell fate specification mechanisms of the cells to reach somatic maturity. This route is inefficient, lengthy and highly variable between cell lines for generation of a single specific cell type for research purposes, and relies on excision of pigmented regions from heterogeneous cultures. However, spontaneous differentiation methods are commonly used for confirmation of pluripotency of a hPSC line for quality assurance.^18^ Knowledge gained through embryology studies of developmental signalling pathways have allowed researchers to develop more focused, sequential, directed differentiation protocols. Directed, or guided, differentiation uses conditioned media containing growth factors or small molecules such as such as Basic Fibroblast Growth Factor (bFGF), the Bone Morphogenetic Protein (BMP) antagonist Noggin, the Wnt antagonist Dickkopf‐1 (DKK1), nicotinamide, casein kinase I inhibitor 7, the ALK4 inhibitor SB‐431542 and the Rho‐associated kinase inhibitor Y‐27632 to modulate specific signalling pathways, mimicking endogenous communication and driving the lineage decision and cell fate of the hPSC.^19^, ^20^, ^21^, ^22^, ^23^ Many protocols have been established for the in vitro directed differentiation of RPE from hESCs^19^, ^21^, ^24^ and iPSCs.^25^, ^26^, ^27^ hPSC derivatives—including RPE cells—are relatively immature, demonstrating a closer similarity to foetal‐like cells than to adult counterparts.^28^ However, they demonstrate characteristics that validate them as valuable models of human adult pathophysiology. In particular, hPSC‐derived RPE cells have consistently been shown to display expression of RPE markers,^29^, ^30^ and key functions observed in native mature RPE cells.^31^, ^32^, ^33^ Importantly, iPSC‐derived RPE retain the complex genetic signatures and variants associated with pathogenesis, making them a valuable tool in disease modelling.

Many retinal dystrophies affect multiple cell types, therefore understanding intracellular networks is fundamental to pathogenesis, which homogeneous two‐dimensional iPSC‐derived culture cannot mimic. Newer methodologies are being developed and optimised to make three‐dimensional in vitro models of the retina called retinal organoids. The laminar retinal organoid allows a further level of complexity to cell culture models.^34^, ^35^ These self‐organising, neural retinas in a dish can be used to interrogate interactions between various cell layers in the retina, and some have even been shown to respond to light.^28^, ^29^ The advantage of this approach is the ability to develop a patient‐derived neural retina in vitro to study the interactions between retinal cell types. Specifically, in cases of retinal disease wherein RPE degeneration results in photoreceptor death, the spatial organisation and temporal signalling between the two cell types could be of importance to the mechanism of disease. Existing retinal organoid protocols produce tissue containing RPE and photoreceptors with rudimentary outer segments which are being developed as a source for potential cell transplantation.^30^ Organoids are a powerful tool in mimicking the physiological structures in affected patients and allow for co‐culture or explant culture of RPE for disease modelling.

## 5 MODELLING INHERITED RETINAL DISEASE

Many blinding diseases manifest by the inherited degeneration of the RPE, and despite the identification of specific mutations causing many of the inherited retinal dystrophies, most of these conditions are currently untreatable. A few examples of inherited retinal diseases are given below, to illustrate how hPSCs can be used for modelling and studying these conditions.

### 5.1 Sorsby fundus dystrophy

Sorsby fundus dystrophy (SFD) is an autosomal dominant IRD caused by variants in the tissue inhibitor of metalloproteinases‐3 (*TIMP3*) gene. TIMP3 is constitutively expressed in the RPE and its monomers and dimers have been found in drusen deposits of SFD eyes. The leading hypothesis to explain the presence of TIMP3 accumulation in SFD eyes was related to a gain or loss of cysteine residues in the TIMP3 variant associated with the disease, resulting in the formation of disulfide bonds and subsequent TIMP3 dimers.^31^ These intramolecular bonds are challenging to break down and since SFD eyes have a diminished ECM turnover rate, it is thought to be a consequence of the dimerisation and an explanation for the accumulation of TIMP3 drusen deposits in the Bruch's Membrane.^31^ Various studies have shown the value of hPSC‐derived RPE cells for the modelling of SFD, with elucidation of key differences in molecular pathways^32^ and retrieval of disease phenotypes observed in vivo, such as drusen‐like deposits.^33^ In comparing iPSC‐derived RPE that have the SFD‐causing variant TIMP3 p.(Ser204Cys) and age‐matched controls lacking the disease‐causing variant, TIMP3 dimers were observed in both SFD and control iPSC‐derived RPE.^32^ These surprising results rejected the commonly held notion that TIMP3 dimerisation drives SFD pathology, instead suggesting excess accumulation of TIMP3, not a functional deficiency in TIMP3, is implicated in ECM changes in SFD.

iPSC studies of SFD have further shown that SFD‐iPSC‐derived RPE cells exhibit diminished trans epithelial electrical resistance, and, as seen in vivo, increased basolateral accumulation of TIMP3.^32^ TIMP3 regulates the thickness of the Bruch's membrane by inhibiting metalloproteinase (MMP) activity,^36^ which catalyse breakdown of the ECM.^31^ Interestingly, no change in TIMP3 expression could be observed across cohorts, and the diseased lines maintained MMP activity.^32^ Proteomic analysis showed enriched expression of cytoskeletal remodelling proteins, and angiogenesis‐related pathways in SFD‐iPSC‐derived RPE.^32^ These results suggest that since MMP activity is retained, the accumulation of active TIMP3 within RPE promotes continuous MMP inhibition, enabling successive ECM synthesis with diminished turnover, resulting in the dysregulated thickening of the BM.

Drusen‐like deposits have also been observed underneath iPSC‐derived RPE cell cultures from both healthy and SFD individuals, yet these were more abundant in the SFD lines and of different composition than in the control lines.^33^ This established the concept that genesis of drusen is RPE‐autonomous, as accumulation of the deposits occurred in the disease model independent of the apically situated photoreceptors or the basal interactions of the innermost capillary structure of the choroid, the choriocapillaris (CC).^33^ It also highlights a quantifiable disease phenotype in vitro that can be used for further understanding of disease pathogenesis. For instance—and in an attempt to understand the role the choriocapillaris plays in macular disease aetiology and in choroidal neovascularization—Manian et al. developed a multicellular model comprised of iPSC‐derived RPE cells and mesenchymal stem cells, a necessity for developing the fenestrated CC‐endothelium, crosslinked with MMP‐degradable peptides to mimic the RPE‐choriocapillaris interface.^37^ This construct allowed the study of locally secreted factors in the progression of choriocapillaris atrophy, a pathology observed in degenerative macular diseases, and described that choroidal neovascularization and choriocapillaris atrophy can be initiated by RPE‐secreted factors exclusively.^37^ Using this model with SFD lines carrying the *TIMP3* disease‐causing variant, the authors showed that choriocapillaris atrophy and choroidal neovascularization at the RPE‐choriocapillaris interface preceded drusen formation and ECM thickening,^37^ which thus brings confirmation on the chronology of SFD progression.

### 5.2 Stargardt disease

Stargardt disease (STGD1) is a progressive macular dystrophy that is genetically heterogeneous with over 90% of cases attributed to a biallelic pathogenic mutation in the *ABCA4* gene that encodes the ATP‐binding cassette, subfamily A, member 4 transporter protein.^38^ In general, ATP‐binding cassette transporters (ABC transporters) are fueled by ATP hydrolysis and translocate substrates across cellular membranes.^39^ Within the normally functioning retina the role of ABCA4 is to work as a retinaldehyde flippase, which facilitates vitamin A shuttling in the photoreceptor outer segments. Specifically, ABCA4 shuttles all‐trans‐retinal from the outer segment disk to the outer segment cytoplasm in the form of retinylidene‐phosphatidylethanolamine (*N*‐retinylidene‐PE). By transporting *N*‐retinylidene‐PE to the cytoplasm side of the disk membrane, it can be dissociated, allowing the released all‐trans‐retinal to enter the visual cycle.^38^ In patient eyes with STGD1, the ABCA4 flippase activity is delayed and the *N*‐retinylidene‐PE lingers long enough in the outer segment to be covalently bound to another retinyl moiety irreversibly forming the cytotoxic, insoluble, bis‐retinoid known as phosphatidylpyridinium bisretinoid (A2PE) and its derivative *N*‐retinyl‐*N*‐retinylidene ethanolamine (A2E). These are then shed in photoreceptor outer segments and phagocytosed by the RPE. A2PE and A2E subsequently accumulate and engorge the RPE causing vermillion fundus. Extensive accumulation appears as yellowish deposits known as pisciform flecks, and the inevitable result is death of the RPE which leads to loss of vision.^38^

*ABCA4* displays extensive allelic heterogeneity with over 1000 pathogenic variants to date.^40^ Various iPSC lines have been generated to represent the myriad of *ABCA4* mutations seen in STGD1.^41^, ^42^ Using iPSC‐derived tissue to model a disease such as STGD1 is useful in attempts to link causality and severity with expression levels, since patient lines retain the complex genetic signatures created via natural splicing and editing that occurs in vivo. In one study, eight iPSC lines from patients harbouring a single pathogenic *ABCA4* variant and normal controls were differentiated into RPE and subjected to RNA equencing and protein analysis.^43^ The resulting principal component analysis of the iPSC‐derived RPE transcriptomes showed the controls clustering distinctly from the patient samples, indicating the differentially expressed genes are driven by *ABCA4* mutations.^43^ In a patient line containing two *ABCA4* splice variants, aberrantly produced polypeptides were short‐lived in the endoplasmic reticulum.^43^ The allele‐specific expression results showed normal *ABCA4* transcripts were present across splice variants, however, the corresponding protein was not detected.^43^ In this study, all patient lines showed upregulation in the unfolded protein response pathway implying membrane mislocalization and protein misfolding, indiscriminate between missense and splice variants.^43^ In comparing patient phenotypes, the higher the missense variant expression, the more deleterious outcomes for the patient, and iPSC studies confirmed this genotype–phenotype correlation in the severity of *ABCA4* variants.^38^, ^43^ Consistent with STGD1 disease phenotypes, the transcriptomic analysis of the patient iPSC‐derived RPE cells, links oxidative and endoplasmic reticulum stress, likely symptoms of reactive responses rather than primary protein alterations due to *ABCA4* variants.^38^, ^43^ This study highlights the benefits of using iPSC‐derived RPE to assess casual variants in difficult to detect genomic regions.

To date, there have been four attempts at cellular intervention in STGD1 patients.^44^, ^45^, ^46^, ^47^ The first clinical phase I/II trials delivered hESC‐derived RPE cell suspension into the region between healthy and atrophic RPE in nine STGD1 patients, who had undergone low‐dose immunosuppression the week prior.^44^ In the patients assessed after 1 year, three showed improvement, no improvement but no decline in three patients, and one patient had vision decline.^44^

### 5.3 Best vitelliform macular dystrophy

Best vitelliform macular dystrophy (Best disease, BD) is an autosomal dominant, early‐onset macular dystrophy frequently caused by mutations in the bestrophin (*BEST1*) gene, with over 200 missense mutations in *BEST1* already identified.^48^ BD is clinically characterised by bilateral vitelliform lesions in the subretinal space in the early stages. In many individuals, these lesions eventually rupture, giving a ‘scrambled egg’ appearance, leading to subfoveal fluid deposition, atrophy of the underlying RPE and progressive irreversible central vision loss. *BEST1* encodes a calcium‐activated chloride channel found in the RPE, with disease‐associated mutations clustering within regions encoding calcium or chloride‐ion binding sites.^49^ BD‐iPSC‐derived RPE cells with various *BEST1* mutations possess various functional defects, including a delayed degradation of POS, increased oxidative stress following chronic POS feeding,^50^, ^51^ defective chloride conductance,^52^ or decreased fluid transport.^53^ Work with BD‐iPSC‐derived RPE has also permitted exploring the potential of gene augmentation to treat BD. Dominantly inherited disorders are commonly excluded as candidates for gene replacement therapies, as it is challenging to silence the dominant allele without altering wild‐type gene expression. Gene augmentation with optional genome editing approaches that allow precision in silencing or repairing pathogenic variants is a powerful solution. In a BD‐iPSC‐derived RPE disease model, gene augmentation restored BEST1 calcium‐activated chloride channel activity in two of the pathogenic model systems, representing two different variants both encoding ion‐binding domains.^48^ A third BD iPSC‐derived RPE model did not respond to the lentiviral constructed gene augmentation, however, the BEST1 channel activity normalised after subsequent CRISPR‐Cas9 editing of the pathogenic variant.^48^ Each of the three rescued models then underwent gene editing to produce premature stops within the pathogenic *BEST1* alleles. Single cell profiling confirmed the RPE transcriptome remained undisturbed.^48^ These results suggest the multipronged approach of personalising gene therapy wherein gene augmentation is used as a first pass and non‐responders become eligible for alternative gene‐editing approaches is a potential strategy for treating BD. iPSC‐RPE model systems like these can be leveraged to maximise safety and efficacy while minimising cost for providing proof of concept gene therapies for impacted individuals. This could be useful and important in ensuring gene therapy development is efficacious and tested on human cells with the genotypic and phenotypic disease profile of interest.

### 5.4 Retinitis pigmentosa

Retinitis pigmentosa (RP) is an irreversible degenerative disease that has been associated with over 40 genes expressed in photoreceptors or the RPE.^54^ The tremendous heterogeneity makes the genetics of RP complex, however, the pathogenesis has been linked to disease‐causing variants in Mer tyrosine kinase (*MERTK*), a gene that encodes a receptor of the Tyro3/Axl/Mer family of tyrosine kinases; and retinoid isomerohydrolase (*RPE65*), a gene that encodes a protein involved in visual transduction post‐light exposure.^55^ Mer tyrosine kinase (MERTK) is expressed by the RPE and critical for phagocytosis of the photoreceptor outer segments.^56^ iPSC studies have compared iPSC‐derived RPE with homozygous or compound heterozygous variants in *MERTK*, and healthy controls. The outcomes showed the cellular morphology remained similar across diseased and control cohorts, however, the engulfment of photoreceptor outer segments was significantly lower in the diseased RPE cells than in controls.^56^

Recessive homozygous disease‐causing variants of *RPE65* occur ~2% of RP cases.^57^ There is a regulatory‐approved *RPE65* retinal gene replacement therapy available for patients suffering from severe RP. However, to be eligible for the therapy, patients must be biallelic for the risk variants, and maintain a specified minimum of uncompromised, functioning retinal cells.^57^ Risk variants in *RPE65* include missense, frameshift, premature stop, in‐frame deletion, and numerous splicing variants.^57^ The onset or severity of RP due to harbouring of the pathogenic *RPE65* allele offers no correlation between genotype and resultant phenotype, providing no link to the location of the causative variants associated. In some patients, the pathogenic *RPE65* mutation, is categorised as a variant of uncertain significance (VUS), regardless of if the phenotype mimics the disease. This disqualifies the patients from RPE65 gene therapy. RPE65 is highly expressed in the RPE, and very minimally in all other tissue types. Due to this fact, iPSC‐derived RPE were generated from those carryingVUS. This allowed bioinformatic interrogation of *RPE65* mRNA in potentially impacted patients to uncover the consequence of this variant and determine the repercussions on retinal disease pathology. Functional studies of RNA expression using the iPSC‐derived RPE confirmed this VUS to be pathogenic thus granting the patient's eligibility for the *RPE65* gene replacement therapy.^57^

Another severe form of RP is called X‐linked retinitis pigmentosa (XLRP), and ~15% of all cases are due to variants in retinitis pigmentosa 2 (RP2) gene.^58^ Retinal organoids obtained from patient‐derived RP2 iPSCs and isogenic knockout controls showed that after 150 days in culture, RP2 patient organoids showed a peak in rod photoreceptor death, and by day 180 there was thinning of the outer nuclear layer. Adeno‐associated virus‐mediated gene amplification with RP2 rescued the outer layer degeneration phenotype in the isogenic controls.^59^ This study provides evidence that retinal organoids can be used as a disease model of retinal degeneration and are a suitable platform for testing potential therapies.

### 5.5 Bietti crystalline dystrophy

Bietti crystalline dystrophy (BCD) is an autosomal recessive inherited retinal disease characterised by crystalline deposits mainly in the macular RPE, resulting in progressive atrophy of the RPE and subsequent deterioration of the connected vasculature the choriocapillaris, and the apically embedded photoreceptors.^60^ A pathogenic variant of *CYP4V2*, a gene belonging to the cytochrome P450 superfamily which encodes a fatty acid hydroxylase, has been identified as causative for BCD.^60^ CYP4V2 is localised most commonly to the endoplasmic reticulum within the RPE and participates in lipid recycling between the RPE and photoreceptors, allowing visual transduction to occur. iPSC‐derived RPE cells from patients carrying the *CYP4V2* mutation exhibit lysosomal dysfunction and impaired autophagy functioning, leading to cellular death.^61^ Mimicking the phenotype seen in postmortem samples from patients with BCD, iPSC‐derived RPE showed degenerative changes such as vacuolated cytoplasm, and accumulation of free cholesterol.^61^ The BCD‐iPSC‐derived RPE disease model associated the accumulation of free cholesterol with lysosomal alkalinization and impairment resulting in dysfunctional RPE autophagy. Introducing cyclodextrins or δ‐tocopherol which reduced free cholesterol, rescued the pathogenic phenotype.^61^ This BD‐iPSC‐derived RPE disease model provides evidence for a therapeutic pathway forward in reducing intracellular cholesterol as a target for BD. Recently, iPSC‐derived RPE cells were also used to assess impact of targeted gene supplementation, with delivery of *CYP4V2* using AAV2 constructs providing functional CYP4V2 to the BD‐iPSC‐derived RPE cells,^62^ and further demonstrating the important and versatile roles hPSC‐derived RPE cells have in preclinical applications.

## 6 MODELLING COMPLEX DISEASE: AGE‐RELATED MACULAR DEGENERATION

Modelling complex diseases such as AMD, require a population‐scale of disease‐specific iPSCs to achieve sufficient statistical power to identify pathogenic variants.^63^, ^64^ Worldwide, evidence of this necessity has been established by several large‐scale initiatives to generate population‐scale, disease specific‐iPSC repositories. Over the past decade, GWAS and whole genome sequencing studies have identified various disease associated variants.^65^ One genetic risk factor, a common variant in the *CFH* gene (*CFH* Y402H, rs1061170 C/C), is estimated to account for nearly half of all AMD risk.^66^, ^67^ Furthermore, variants at the *LOC387715/ARMS2/HTRA1* (rs10490924 T/T) locus have been identified as major contributors to AMD development.^68^, ^69^ To date, genome‐wide association studies (GWAS) have identified over 30 independent loci where a common risk allele is associated with an increased risk of AMD.^70^, ^71^, ^72^ These loci influence distinct biological pathways, such as the complement system, lipid transport, extracellular matrix remodelling, angiogenesis and cell survival.^73^ In an attempt to provide a population‐scale approach to iPSC‐disease modelling for complex disease, we recently described both transcriptomic and proteomic profiles of iPSC‐derived RPE cells from a large cohort of people with or without the advanced form of AMD geographic atrophy.^74^ The comparison of single cell RNA sequencing from iPSC‐derived RPE cells of 43 patients at the phenotypic end of AMD and of 36 unaffected controls implicated genes at loci definitively associated with disease such as the *CFH* and *ARMS2*/*HTRA1* loci, and also uncovered new pathogenic loci and pathways.^74^ In particular, mitochondrial pathways were identified as dysregulated in geographic atrophy RPE cells, data confirmed by mass spectrometry and by functional analysis of mitochondrial respiration.^74^

Other studies using small subsets of hPSCs have also identified phenotypes associated with AMD. iPSC‐RPE cells derived from patients with AMD compared to healthy controls display a reduced activity of the mitochondrial antioxidant enzyme superoxide dismutase 2 (SOD2) and have a higher susceptibility to oxidative stress.^75^, ^76^, ^77^, ^78^ Additionally, AMD iPSC‐derived RPE cells exhibit a decreased autophagy and complement dysregulation.^75^, ^76^, ^77^ A hallmark of AMD is the accumulation of sub‐RPE drusen‐like deposits of lipids and proteins, and RPE degeneration follows soon after. Evidence of an adequate AMD iPSC‐derived RPE disease model is presence of drusen. Drusen components such as apolipoprotein E (APOE),^79^ vitronectin,^80^ and TIMP3,^81^ have been shown to accumulate in AMD disease models using iPSC‐derived RPE from affected patients.^33^, ^81^, ^82^ These in vitro studies have demonstrated that drusen‐like deposits can accumulate with a homogeneous population of RPE. Drusen‐like material has been observed by 3 months in culture of iPSC‐derived RPE, and was more frequent and dense when RPE was derived from AMD patients.^33^ It has been shown that by co‐culturing with retinal progenitor cells (RPCs), large mounds of drusen‐like deposits appear that functionally impact permeability,^81^ thus mimicking the damaged Bruch's Membrane seen in AMD.

Drug discovery and screening using iPSCs provides the possibility of scalable, personalised therapies for patients suffering from any genetic disorder,^65^ including retinal disease. One study used a panel of AMD biomarkers for a candidate drug screen, and in combination with transcriptome analysis, uncovered that the vitamin B3 derivative nicotinamide is a promising avenue for interrogation. After showing the iPSC‐derived RPE produced drusen‐related proteins, with the *ARMS2/HTRA1* AMD donor lines showing the most pronounced accumulation, the study found that nicotinamide ameliorated AMD‐related phenotypes. It is known that ARMS2/HTRA1 lines have a compromised superoxide dismutase 2 response, implying increased vulnerability to oxidative mitochondrial stress.^75^ Nicotinamide has antioxidant and anti‐inflammatory properties^83^ that protect mitochondria against reactive oxygen species (ROS) and excessive fragmentation by decreasing mitochondrial protein acetylation^84^ via upregulation of SIRT‐1 a known chromatin modifying gene^85^. Nicotinamide inhibits drusen proteins and complement factors while upregulating nucleosome, ribosome, and chromatin‐modifying genes. This study provides evidence towards developing therapeutics that target nicotinamide‐regulated pathways.^77^ More recently, a study used iPSC‐derived RPE from AMD patients to test the efficacy of three mitochondrial maintenance drugs: AICAR (5‐Aminoimidazole‐4‐carboxamide ribonucleotide), Metformin, and Trehalose. After acute and extended drug exposure, the mitochondria of the RPE were analysed and the results showed differential responses to the drugs across patient lines.^86^ This study shows the need for a personalised medicinal approach, and supports the feasibility of using iPSC‐derived RPE to develop a personalised drug treatment plan for patients with AMD.

Finally, iPSCs have made the concept of autologous cellular replacement therapy a reality. Autologous cell replacement therapy minimises the risk of immune rejection in patients because the cells are derived from the patient themself rather than a donor.^87^ The eye is an ideal organ for stem cell based therapies due to its accessibility and the advent of high resolution in vivo retinal imaging such as optical tomography which allows for unambiguous and continuous monitoring.^88^ Clinical trials have commenced wherein patient‐derived iPSCs are differentiated into RPE and surgically implanted into the damaged retina of the patients.^89^, ^90^, ^91^, ^92^, ^93^ Current stem cell therapies cater to outer retinal degeneration of the RPE and photoreceptors to treat AMD and RP. hPSC‐derived RPE as cell suspension or monolayer sheets (with or without scaffolds) have been used to attempt to halt the disease progression of AMD by cellular transplantation and restore patient vision.^89^, ^90^, ^91^, ^92^, ^93^ In comparison with RPE cell suspension, RPE sheet transplantation is more surgically invasive due to the need to create a larger incision and on occasion requires removal of choroidal neovascularization prior to insertion of the sheet. Retinal haemorrhage, edema and other adverse effects have been reported as outcomes of the RPE sheet surgery.^89^, ^90^, ^91^ Outcomes of RPE sheet transplantation are promising and show either unchanged or improvement in visual acuity with no evidence of worsening vision.^89^, ^90^, ^91^, ^92^, ^93^ There are predictions that RPE sheet transplantation could have implications long‐term and severity could parallel scaffold composition, as two clinical trials used non‐biodegradable scaffolds.^88^ However, studies have confirmed that a subretinally transplanted autologous iPSC‐derived RPE sheet can survive below the retina for at least 4 years with no adverse events.^92^ Transplantation using RPE cell suspension is less invasive than sheets and there are no adverse consequences associated with surgical outcomes. However, adverse events reported with RPE cell suspension transplantation were associated with side effects of immunosuppressants such as mycophenolate mofetil and tacrolimus,^44^, ^45^, ^94^ although recent reports suggest that long term immunosuppression may not be required.^95^ There were reports of a thin layer of tissue forming on the retina referred to as an epiretinal membrane, which was attributed to cellular leakage from the site of transplant.^44^, ^45^, ^94^, ^96^ Unfortunately to date this therapy shows meagre outcomes with minimal improvement in retinal sensitivity but no statistically significant increase in visual acuity or patient reported quality of life.^44^, ^45^, ^94^, ^96^

## 7 CONCLUSION

There is a pressing and continuous need for human models of disease to improve our understanding of retinal degenerative diseases. hPSC‐derived RPE, and retinal organoid models containing RPE are powerful tools for elucidating underlying disease genotypes and pathological phenotypes, developing clinically suitable therapeutics for treatment of IRDs and more complex conditions such as AMD. The genome editing technology which takes advantage of a bacterial immune system, clustered regularly interspaced short palindromic repeat (CRISPR)‐associated protein (Cas) and an RNA that guides the Cas protein to a specific location in the genome has emerged as a precise tool for correcting monogenic IRDs.^97^ CRISPR‐based genome editing allows for permanent replacement or repair of causal genetic variants, and shows promise in treating complex retinal degenerative diseases such as AMD. An in vivo editing vector has been developed to induce permanent inhibition of VEGF via CRISPR‐mediated knockout in the RPE, which could prevent further neovascularization in AMD patients.^98^ Furthermore, in a human embryonic kidney cell line a CRISPR/Cas‐mediated strategy has been shown to correct an allele conferring AMD risk in the complement factor H gene.^99^ Although in vivo CRISPR‐based agents have been used to gene correct adults with congenital blindness,^100^ the attractiveness of the therapeutic potential of precise genome modifications is often overshadowed by the safety aspects associated with the CRISPR/Cas technology. Marrying CRISPR/Cas and iPSC technology is an important tool for interrogating safety and efficacy of cellular therapeutics by improving delivery methods, promoting detection of rare variants, and discovering off‐target effects. This technology is paving the way forward for personalised therapeutics, drug discovery, and cellular therapy at scale. It is however important to acknowledge the limitations associated with hPSC disease models and to highlight where these models are most valuable. For instance, a concern for using iPSCs to study complex conditions such as AMD, where genetics, age and environment play roles, is that iPSC‐derived RPE have a transcriptional profile that resembles foetal cells^101^ and do not live a lifetime of molecular events as their native counterpart would, which must still be carefully considered when interpreting data. Furthermore, in vivo, cells do not live in autonomy, but form parts of tissues and organs with intercellular exchanges and communications, a complexity that is not yet reproduced in current in vitro models. The development of better models could incorporate ‘*on demand*’ levels of maturity of cell populations, layering of various cell populations in set numbers, from the same embryonic lineage or other lineages (such as endothelial cells and immune cells), and in environmental conditions closer to their native counterpart. Much effort is already dedicated to developing these complex models^102^ that will undoubtedly provide powerful new tools in the fight against vision loss and blindness.

## FUNDING INFORMATION

This research was supported by the Australian Research Council (ARC) Training Centre for Personalised Therapeutics Technologies (IC170100016, JH, AP), a National Health and Medical Research Council (NHMRC) Senior Research Fellowship (AP, 1154389), a NHMRC Synergy grant (1181010, AP), the DHB Foundation (GEL, AP), the Medical Research Future Fund ‐ Stem Cell Therapies Mission (GEL, AP, MRF1200678), Dementia Australia Research Foundation (039305, GEL); the University of Melbourne and Operational Infrastructure support from the Victorian Government.

## CONFLICT OF INTEREST

The authors declare no conflict of interest.

## References

[1] Takahashi K , Tanabe K , Ohnuki M , et al. Induction of pluripotent stem cells from adult human fibroblasts by defined factors. Cell. 2007;131:861‐872. 18035408 10.1016/j.cell.2007.11.019

[2] Tan LX , Germer CJ , Cunza NL , Lakkaraju A . Complement activation, lipid metabolism, and mitochondrial injury: converging pathways in age‐related macular degeneration. Redox Biol. 2020;37:101781. 33162377 10.1016/j.redox.2020.101781 PMC7767764

[3] Ahmad I , Teotia P , Erickson H , Xia X . Recapitulating developmental mechanisms for retinal regeneration. Prog Retin Eye Res. 2019;76:100824. 31843569 10.1016/j.preteyeres.2019.100824 PMC12866890

[4] Marmorstein AD . The polarity of the retinal pigment epithelium. Traffic. 2001;2:867‐872. 11737824 10.1034/j.1600-0854.2001.21202.x

[5] Pugazhendhi A , Hubbell M , Jairam P , Ambati B . Neovascular macular degeneration: a review of etiology, risk factors, and recent advances in research and therapy. Int J Mol Sci. 2021;22:1170. 33504013 10.3390/ijms22031170 PMC7866170

[6] Simó R , Villarroel M , Corraliza L , Hernández C , Garcia‐Ramírez M . The retinal pigment epithelium: something more than a constituent of the blood‐retinal barrier—implications for the pathogenesis of diabetic retinopathy. J Biomed Biotechnol. 2010;2010:190724. 20182540 10.1155/2010/190724 PMC2825554

[7] Levkovitch‐Verbin H . Animal models of optic nerve diseases. Eye. 2004;18:1066‐1074. 15534591 10.1038/sj.eye.6701576

[8] Paigen K . A miracle enough: the power of mice. Nat Med. 1995;1:215‐220. 7585036 10.1038/nm0395-215

[9] Lo B , Parham L . Ethical issues in stem cell research. Endocr Rev. 2009;30:204‐213. 19366754 10.1210/er.2008-0031 PMC2726839

[10] Waterston RH , Lander ES , Sulston JE . On the sequencing of the human genome. Proc Natl Acad Sci. 2002;99:3712‐3716. 11880605 10.1073/pnas.042692499 PMC122589

[11] Fronk AH , Vargis E . Methods for culturing retinal pigment epithelial cells: a review of current protocols and future recommendations. J Tissue Eng. 2016;7:2041731416650838. 27493715 10.1177/2041731416650838 PMC4959307

[12] Shang P , Stepicheva NA , Hose S , Zigler JS , Sinha D . Primary cell cultures from the mouse retinal pigment epithelium. J Vis Exp. 2018;16(133):56997. doi:10.3791/56997 PMC5931770 29608155

[13] Sterneckert JL , Reinhardt P , Schöler HR . Investigating human disease using stem cell models. Nat Rev Genet. 2014;15:625‐639. 25069490 10.1038/nrg3764

[14] Kim D , Kim CH , Moon JI , et al. Generation of human induced pluripotent stem cells by direct delivery of reprogramming proteins. Cell Stem Cell. 2009;4:472‐476. 19481515 10.1016/j.stem.2009.05.005 PMC2705327

[15] Warren L , Manos PD , Ahfeldt T , et al. Highly efficient reprogramming to pluripotency and directed differentiation of human cells with synthetic modified mRNA. Cell Stem Cell. 2010;7:618‐630. 20888316 10.1016/j.stem.2010.08.012 PMC3656821

[16] Subramanyam D , Lamouille S , Judson RL , et al. Multiple targets of miR‐302 and miR‐372 promote reprogramming of human fibroblasts to induced pluripotent stem cells. Nat Biotechnol. 2011;29:443‐448. 21490602 10.1038/nbt.1862 PMC3685579

[17] Teo AKK , Arnold SJ , Trotter MWB , et al. Pluripotency factors regulate definitive endoderm specification through eomesodermin. Gene Dev. 2011;25:238‐250. 21245162 10.1101/gad.607311 PMC3034899

[18] Allison TF , Andrews PW, Avior Y, et al. Assessment of established techniques to determine developmental and malignant potential of human pluripotent stem cells. Nat Commun. 2018;9:1925. 29765017 10.1038/s41467-018-04011-3 PMC5954055

[19] Buchholz DE , Pennington BO , Croze RH , Hinman CR , Coffey PJ , Clegg DO . Rapid and efficient directed differentiation of human pluripotent *stem cells Int*o retinal pigmented epithelium. Stem Cell Transl Med. 2013;2:384‐393. 10.5966/sctm.2012-0163 PMC3667566 23599499

[20] Idelson M , Alper R , Obolensky A , et al. Directed differentiation of human embryonic stem cells into functional retinal pigment epithelium cells. Cell Stem Cell. 2009;5:396‐408. 19796620 10.1016/j.stem.2009.07.002

[21] Osakada F , Jin ZB , Hirami Y , et al. In vitro differentiation of retinal cells from human pluripotent stem cells by small‐molecule induction. J Cell Sci. 2009;122:3169‐3179. 19671662 10.1242/jcs.050393

[22] Reichman S , Terray A , Slembrouck A , et al. From confluent human iPS cells to self‐forming neural retina and retinal pigmented epithelium. Proc Natl Acad Sci. 2014;111:8518‐8523. 24912154 10.1073/pnas.1324212111 PMC4060726

[23] Kuwahara A , Ozone C , Nakano T , Saito K , Eiraku M , Sasai Y . Generation of a ciliary margin‐like stem cell niche from self‐organizing human retinal tissue. Nat Commun. 2015;6:6286. 25695148 10.1038/ncomms7286

[24] Lidgerwood GE , Lim SY , Crombie DE , et al. Defined medium conditions for the induction and expansion of human pluripotent stem cell‐derived retinal pigment epithelium. Stem Cell Rev Rep. 2016;12:179‐188. 26589197 10.1007/s12015-015-9636-2

[25] D'Antonio‐Chronowska A , D'Antonio M , Frazer K . In vitro differentiation of human iPSC‐derived retinal pigment epithelium cells (iPSC‐RPE). Bio‐protocol. 2019;9:e3469. 33654959 10.21769/BioProtoc.3469 PMC7853967

[26] Hazim RA , Karumbayaram S , Jiang M , et al. Differentiation of RPE cells from integration‐free iPS cells and their cell biological characterization. Stem Cell Res Ther. 2017;8:217. 28969679 10.1186/s13287-017-0652-9 PMC5625837

[27] Sharma R , Khristov V , Rising A , et al. Clinical‐grade stem cell–derived retinal pigment epithelium patch rescues retinal degeneration in rodents and pigs. Sci Transl Med. 2019;11:eaat5580. 30651323 10.1126/scitranslmed.aat5580 PMC8784963

[28] Meyer JS et al. Optic vesicle‐like structures derived from human pluripotent stem cells facilitate a customized approach to retinal disease treatment. Stem Cells. 2011;29:1206‐1218. 21678528 10.1002/stem.674 PMC3412675

[29] Hallam D , Hilgen G , Dorgau B , et al. Human‐induced pluripotent stem cells generate light responsive retinal organoids with variable and nutrient‐dependent efficiency. Stem Cells. 2018;36:1535‐1551. 30004612 10.1002/stem.2883 PMC6392112

[30] Hau K‐L , Lane A , Guarascio R , Cheetham ME . Eye on a dish models to evaluate splicing modulation. Methods Mol Biol Clifton N J. 2022;2434:245‐255. 10.1007/978-1-0716-2010-6_16 PMC9703233 35213022

[31] Christensen DRG , Brown FE , Cree AJ , Ratnayaka JA , Lotery AJ . Sorsby fundus dystrophy—a review of pathology and disease mechanisms. Exp Eye Res. 2017;165:35‐46. 28847738 10.1016/j.exer.2017.08.014

[32] Hongisto H , Dewing JM , Christensen DRG , et al. In vitro stem cell modelling demonstrates a proof‐of‐concept for excess functional mutant TIMP3 as the cause of Sorsby fundus dystrophy. J Pathol. 2020;252:138‐150. 32666594 10.1002/path.5506

[33] Galloway CA , Dalvi S , Hung SSC , et al. Drusen in patient‐derived hiPSC‐RPE models of macular dystrophies. Proc Natl Acad Sci. 2017;114:E8214‐E8223. 28878022 10.1073/pnas.1710430114 PMC5625924

[34] Nakano T , Ando S , Takata N , et al. Self‐formation of optic cups and storable stratified neural retina from human ESCs. Cell Stem Cell. 2012;10:771‐785. 22704518 10.1016/j.stem.2012.05.009

[35] Mellough CB , Collin J , Queen R , et al. Systematic comparison of retinal organoid differentiation from human pluripotent stem cells reveals stage specific, cell line, and methodological differences. Stem Cell Transl Med. 2019;8:694‐706. 10.1002/sctm.18-0267 PMC6591558 30916455

[36] Ruiz A , Brett P , Bok D . TIMP‐3 is expressed in the human retinal pigment epithelium. Biochem Bioph Res Co. 1996;226:467‐474. 10.1006/bbrc.1996.1379 8806658

[37] Manian KV , Galloway CA , Dalvi S , et al. 3D iPSC modeling of the retinal pigment epithelium‐choriocapillaris complex identifies factors involved in the pathology of macular degeneration. Cell Stem Cell. 2021;28:846‐862.e8. doi:10.1016/j.stem.2021.02.006 33784497 PMC8520418

[38] Braun TA , Mullins RF , Wagner AH , et al. Non‐exomic and synonymous variants in ABCA4 are an important cause of Stargardt disease. Hum Mol Genet. 2013;22:5136‐5145. 23918662 10.1093/hmg/ddt367 PMC3842174

[39] Tsybovsky Y , Molday RS , Palczewski K . The ATP‐binding cassette transporter ABCA4: structural and functional properties and role in retinal disease. Adv Exp Med Biol. 2010;703:105‐125. 20711710 10.1007/978-1-4419-5635-4_8 PMC2930353

[40] Cornelis SS , Bax NM , Zernant J , et al. In silico functional meta‐analysis of 5,962 ABCA4 variants in 3,928 retinal dystrophy cases. Hum Mutat. 2017;38:400‐408. 28044389 10.1002/humu.23165

[41] Claassen JN , Zhang D , Chen SC , et al. Generation of the induced pluripotent stem cell line from a patient with autosomal recessive ABCA4‐mediated Stargardt macular dystrophy. Stem Cell Res. 2018;34:101352. 30634128 10.1016/j.scr.2018.11.013

[42] Riera M , Patel A , Burés‐Jelstrup A , et al. Generation of two iPS cell lines (FRIMOi003‐a and FRIMOi004‐a) derived from Stargardt patients carrying ABCA4 compound heterozygous mutations. Stem Cell Res. 2019;36:101389. 30798147 10.1016/j.scr.2019.101389 PMC6668028

[43] Matynia A , Wang J , Kim S , et al. Assessing variant causality and severity using retinal pigment epithelial cells derived from Stargardt disease patients. Transl Vis Sci Technol. 2022;11:33. 10.1167/tvst.11.3.33 PMC8976924 35348597

[44] Schwartz SD , Regillo CD , Lam BL , et al. Human embryonic stem cell‐derived retinal pigment epithelium in patients with age‐related macular degeneration and Stargardt's macular dystrophy: follow‐up of two open‐label phase 1/2 studies. Lancet. 2015;385:509‐516. 25458728 10.1016/S0140-6736(14)61376-3

[45] Mehat MS , Sundaram V , Ripamonti C , et al. Transplantation of human embryonic stem cell‐derived retinal pigment epithelial cells in macular degeneration. Ophthalmology. 2018;125:1765‐1775. 29884405 10.1016/j.ophtha.2018.04.037 PMC6195794

[46] Li S et al. A phase I clinical trial of human embryonic stem cell‐derived retinal pigment epithelial cells for early‐stage Stargardt macular degeneration: 5‐years' follow‐up. Cell Proliferat. 2021;54:e13100. 10.1111/cpr.13100 PMC8450131 34347352

[47] Sung Y , Lee MJ , Choi J , et al. Long‐term safety and tolerability of subretinal transplantation of embryonic stem cell‐derived retinal pigment epithelium in Asian Stargardt disease patients. Brit J Ophthalmol. 2021;105:829‐837. 32727729 10.1136/bjophthalmol-2020-316225

[48] Sinha D , Steyer B , Shahi PK , et al. Human iPSC modeling reveals mutation‐specific responses to gene therapy in a genotypically diverse dominant maculopathy. Am J Hum Genet. 2020;107:278‐292. 32707085 10.1016/j.ajhg.2020.06.011 PMC7413860

[49] Dickson VK , Pedi L , Long SB . Structure and insights into the function of a Ca^2+^‐activated Cl^−^ channel. Nature. 2014;516:213‐218. 25337878 10.1038/nature13913 PMC4454446

[50] Singh R , Shen W , Kuai D , et al. iPS cell modeling of best disease: insights into the pathophysiology of an inherited macular degeneration. Hum Mol Genet. 2013;22:593‐607. 23139242 10.1093/hmg/dds469 PMC3542866

[51] Singh R , Kuai D , Guziewicz KE , et al. Pharmacological modulation of photoreceptor outer segment degradation in a human iPS cell model of inherited macular degeneration. Mol Ther. 2015;23:1700‐1711. 26300224 10.1038/mt.2015.141 PMC4817951

[52] Moshfegh Y , Velez G , Li Y , Bassuk AG , Mahajan VB , Tsang SH . BESTROPHIN1 mutations cause defective chloride conductance in patient stem cell‐derived RPE. Hum Mol Genet. 2016;25:ddw126‐dd2680. doi:10.1093/hmg/ddw126 PMC5181636 27193166

[53] Lee JH , Oh J , Lee CS . Induced pluripotent stem cell modeling of best disease and autosomal recessive bestrophinopathy. Yonsei Med J. 2020;61:816‐825. 32882766 10.3349/ymj.2020.61.9.816 PMC7471084

[54] Ferrari S , di Iorio E , Barbaro V , Ponzin D , Sorrentino FS , Parmeggiani F . Retinitis pigmentosa: genes and disease mechanisms. Curr Genom. 2011;12:238‐249. 10.2174/138920211795860107 PMC3131731 22131869

[55] Sparrrow JR , Hicks D , Hamel CP . The retinal pigment epithelium in health and disease. Curr Mol Med. 2010;10:802‐823. 21091424 10.2174/156652410793937813 PMC4120883

[56] Tagawa M , Ikeda HO , Inoue Y , et al. Deterioration of phagocytosis in induced pluripotent stem cell‐derived retinal pigment epithelial cells established from patients with retinitis pigmentosa carrying Mer tyrosine kinase mutations. Exp Eye Res. 2021;205:108503. 33609509 10.1016/j.exer.2021.108503

[57] Nash BM , Loi TH , Fernando M , et al. Evaluation for retinal therapy for RPE65 variation assessed in hiPSC retinal pigment epithelial cells. Stem Cells Int. 2021;2021:4536382. 34938339 10.1155/2021/4536382 PMC8687838

[58] Breuer DK , Yashar BM , Filippova E , et al. A comprehensive mutation analysis of RP2 and RPGR in a North American cohort of families with X‐linked retinitis pigmentosa. Am J Hum Genet. 2002;70:1545‐1554. 11992260 10.1086/340848 PMC379141

[59] Lane A , Jovanovic K , Shortall C , et al. Modeling and rescue of RP2 retinitis Pigmentosa using iPSC‐derived retinal organoids. Stem Cell Rep. 2020;15:67‐79. 10.1016/j.stemcr.2020.05.007 PMC7363745 32531192

[60] Ng DSC , Lai TYY , Ng TK , Pang CP . Genetics of Bietti crystalline dystrophy. Asia‐Pac J Ophthalmol. 2016;5:245‐252. 10.1097/APO.0000000000000209 27228076

[61] Hata M , Ikeda HO , Iwai S , et al. Reduction of lipid accumulation rescues Bietti's crystalline dystrophy phenotypes. Proc Natl Acad Sci USA. 2018;115:3936‐3941. 29581279 10.1073/pnas.1717338115 PMC5899444

[62] Wang JH , Lidgerwood GE , Daniszewski M , et al. AAV2‐mediated gene therapy for Bietti crystalline dystrophy provides functional CYP4V2 in multiple relevant cell models. Sci Rep. 2022. doi:10.1038/s41598-022-12210-8 PMC9184470 35680963

[63] Soldner F , Jaenisch R . Stem cells, genome editing, and the path to translational medicine. Cell. 2018;175:615‐632. 30340033 10.1016/j.cell.2018.09.010 PMC6461399

[64] Guo MH , Dauber A , Lippincott MF , Chan YM , Salem RM , Hirschhorn JN . Determinants of power in gene‐based burden testing for monogenic disorders. Am J Hum Genet. 2016;99:527‐539. 27545677 10.1016/j.ajhg.2016.06.031 PMC5011058

[65] Elitt MS , Barbar L , Tesar PJ . Drug screening for human genetic diseases using iPSC models. Hum Mol Genet. 2018;27:R89‐R98. 29771306 10.1093/hmg/ddy186 PMC6061782

[66] Hageman GS , Anderson DH , Johnson LV , et al. A common haplotype in the complement regulatory gene factor H (HF1/CFH) predisposes individuals to age‐related macular degeneration. Proc Natl Acad Sci USA. 2005;102:7227‐7232. 15870199 10.1073/pnas.0501536102 PMC1088171

[67] Klein RJ , Zeiss C , Chew EY , et al. Complement factor H polymorphism in age‐related macular degeneration. Science. 2005;308:385‐389. 15761122 10.1126/science.1109557 PMC1512523

[68] Rivera A , Fisher SA , Fritsche LG , et al. Hypothetical LOC387715 is a second major susceptibility gene for age‐related macular degeneration, contributing independently of complement factor H to disease risk. Hum Mol Genet. 2005;14:3227‐3236. 16174643 10.1093/hmg/ddi353

[69] Jakobsdottir J et al. Susceptibility genes for age‐related maculopathy on chromosome 10q26. Am J Hum Genet. 2005;77:389‐407. 16080115 10.1086/444437 PMC1226205

[70] Fritsche LG , Igl W , Bailey JNC , et al. A large genome‐wide association study of age‐related macular degeneration highlights contributions of rare and common variants. Nat Genet. 2016;48:134‐143. 26691988 10.1038/ng.3448 PMC4745342

[71] Yan Q , Ding Y , Liu Y , et al. Genome‐wide analysis of disease progression in age‐related macular degeneration. Hum Mol Genet. 2018;27:929‐940. 29346644 10.1093/hmg/ddy002 PMC6059197

[72] Han X , Gharahkhani P , Mitchell P , Liew G , Hewitt AW , MacGregor S . Genome‐wide meta‐analysis identifies novel loci associated with age‐related macular degeneration. J Hum Genet. 2020;65:657‐665. 32277175 10.1038/s10038-020-0750-x

[73] Fritsche LG et al. Seven new loci associated with age‐related macular degeneration. Nat Genet. 2013;45:433‐439. 23455636 10.1038/ng.2578 PMC3739472

[74] Senabouth A et al. Transcriptomic and proteomic retinal pigment epithelium signatures of age‐related macular degeneration. bioRxiv. 2021. doi:10.1101/2021.08.19.457044 PMC9325891 35882847

[75] Yang J , Li Y , Chan L , et al. Validation of genome‐wide association study (GWAS)‐identified disease risk alleles with patient‐specific stem cell lines. Hum Mol Genet. 2014;23:3445‐3455. 24497574 10.1093/hmg/ddu053 PMC4049304

[76] Hallam D , Collin J , Bojic S , et al. An induced pluripotent stem cell patient specific model of complement factor H (Y402H) polymorphism displays characteristic features of age‐related macular degeneration and indicates a beneficial role for UV light exposure. Stem Cells Dayt Ohio. 2017;35:2305‐2320. 10.1002/stem.2708 PMC5698780 28913923

[77] Saini JS , Corneo B , Miller JD , et al. Nicotinamide ameliorates disease phenotypes in a human iPSC model of age‐related macular degeneration. Cell Stem Cell. 2017;20:635‐647.e7. 28132833 10.1016/j.stem.2016.12.015 PMC5419856

[78] Golestaneh N , Chu Y , Cheng SK , Cao H , Poliakov E , Berinstein DM . Repressed SIRT1/PGC‐1α pathway and mitochondrial disintegration in iPSC‐derived RPE disease model of age‐related macular degeneration. J Transl Med. 2016;14:344. 27998274 10.1186/s12967-016-1101-8 PMC5175395

[79] Johnson LV , Forest DL , Banna CD , et al. Cell culture model that mimics drusen formation and triggers complement activation associated with age‐related macular degeneration. Proc Natl Acad Sci. 2011;108:18277‐18282. 21969589 10.1073/pnas.1109703108 PMC3215058

[80] Hageman GS , Mullins RF . Molecular composition of drusen as related to substructural phenotype. Mol Vis. 1999;5:28. 10562652

[81] Chen X , Singh D , Adelman RA , Rizzolo LJ . Unstimulated, serum‐free cultures of retinal pigment epithelium excrete large mounds of Drusen‐like deposits. Curr Eye Res. 2020;45:1390‐1394. 32202447 10.1080/02713683.2020.1740744

[82] Engel AL , Wang YK , Khuu TH , et al. Extracellular matrix dysfunction in Sorsby patient‐derived retinal pigment epithelium. Exp Eye Res. 2022;215:108899. 34929159 10.1016/j.exer.2021.108899 PMC8923943

[83] Maiese K , Chong ZZ , Hou J , Shang YC . The vitamin nicotinamide: translating nutrition into clinical care. Molecules. 2009;14:3446‐3485. 19783937 10.3390/molecules14093446 PMC2756609

[84] Klimova N , Long A , Kristian T . Nicotinamide mononucleotide alters mitochondrial dynamics by SIRT3‐dependent mechanism in male mice. J Neurosci Res. 2019;97:975‐990. 30801823 10.1002/jnr.24397 PMC6565489

[85] Jang S , Kang HT , Hwang ES . Nicotinamide‐induced mitophagy. J Biol Chem. 2012;287:19304‐19314. 22493485 10.1074/jbc.M112.363747 PMC3365962

[86] Ebeling MC , Geng Z , Stahl MR , et al. Testing mitochondrial‐targeted drugs in iPSC‐RPE from patients with age‐related macular degeneration. Pharm. 2022;15:62. 10.3390/ph15010062 PMC8781759 35056119

[87] Yamanaka S . Strategies and new developments in the generation of patient‐specific pluripotent stem cells. Cell Stem Cell. 2007;1:39‐49. 18371333 10.1016/j.stem.2007.05.012

[88] Maeda T , Mandai M , Sugita S , Kime C , Takahashi M . Strategies of pluripotent stem cell‐based therapy for retinal degeneration: update and challenges. Trends Mol Med. 2022;28:388‐404. doi:10.1016/j.molmed.2022.03.001 35370091

[89] Kashani AH , Lebkowski JS , Rahhal FM , et al. A bioengineered retinal pigment epithelial monolayer for advanced, dry age‐related macular degeneration. Sci Transl Med. 2018;10(435). doi:10.1126/scitranslmed.aao4097 29618560

[90] Kashani AH , Lebkowski JS , Rahhal FM , et al. One‐year follow‐up in a phase 1/2a clinical trial of an allogeneic RPE cell bioengineered implant for advanced dry age‐related macular degeneration. Transl Vis Sci Technol. 2021;10:13. 10.1167/tvst.10.10.13 PMC8496407 34613357

[91] da Cruz L et al. Phase 1 clinical study of an embryonic stem cell–derived retinal pigment epithelium patch in age‐related macular degeneration. Nat Biotechnol. 2018;36:328‐337. 29553577 10.1038/nbt.4114

[92] Takagi S , Mandai M , Gocho K , et al. Evaluation of transplanted autologous induced pluripotent stem cell‐derived retinal pigment epithelium in exudative age‐related macular degeneration. Ophthalmol Retin. 2019;3:850‐859. 10.1016/j.oret.2019.04.021 31248784

[93] Mandai M , Watanabe A , Kurimoto Y , et al. Autologous induced stem‐cell–derived retinal cells for macular degeneration. New Engl J Med. 2017;376:1038‐1046. 28296613 10.1056/NEJMoa1608368

[94] Song WK , Park KM , Kim HJ , et al. Treatment of macular degeneration using embryonic stem cell‐derived retinal pigment epithelium: preliminary results in Asian patients. Stem Cell Rep. 2015;4:860‐872. 10.1016/j.stemcr.2015.04.005 PMC4437471 25937371

[95] Kashani AH , Lebkowski JS , Hinton DR , et al. Survival of an HLA‐mismatched, bioengineered RPE implant in dry age‐related macular degeneration. Stem Cell Rep. 2022;17:448‐458. 10.1016/j.stemcr.2022.01.001 PMC9039755 35120620

[96] Sugita S , Mandai M , Hirami Y , et al. HLA‐matched allogeneic iPS cells‐derived RPE transplantation for macular degeneration. J Clin Med. 2020;9:2217. 10.3390/jcm9072217 PMC7408794 32668747

[97] Benati D , Patrizi C , Recchia A . Gene editing prospects for treating inherited retinal diseases. J Med Genet. 2020;57:437‐444. 31857428 10.1136/jmedgenet-2019-106473

[98] Kim E , Koo T , Park SW , et al. In vivo genome editing with a small Cas9 orthologue derived from *Campylobacter jejuni* . Nat Commun. 2017;8:14500. 28220790 10.1038/ncomms14500 PMC5473640

[99] Tran MTN , Khalid MKNM , Pébay A , et al. Screening of CRISPR/Cas base editors to target the AMD high‐risk Y402H complement factor H variant. Mol Vis. 2019;25:174‐182. 30996586 PMC6441356

[100] Harrison C . First CRISPR therapy dosed. Nat Biotechnol. 2020;38:382. 10.1038/s41587-020-0493-4 32265555

[101] Peng S , Gan G , Qiu C , et al. Engineering a blood‐retinal barrier with human embryonic stem cell‐derived retinal pigment epithelium: transcriptome and functional analysis. Stem Cell Transl Med. 2013;2:534‐544. 10.5966/sctm.2012-0134 PMC3697821 23734062

[102] Lidgerwood GE , Hewitt AW , Pébay A . Human pluripotent stem cells for the modelling of diseases of the retina and optic nerve: toward a retina in a dish. Curr Opin Pharmacol. 2019;48:114‐119. 31590110 10.1016/j.coph.2019.09.003

